# Quantitative assessment of the association between MHTFR C677T (rs1801133, Ala222Val) polymorphism and susceptibility to bladder cancer

**DOI:** 10.1186/1746-1596-8-95

**Published:** 2013-06-17

**Authors:** Wei Xu, Haifeng Zhang, Fa Wang, Honghui Wang

**Affiliations:** 1 Department of Interventional Radiology, The Second Affiliated Hospital of Harbin Medical University, Harbin 150086, China; 2 Department of Urinary Surgery, The Second Affiliated Hospital of Harbin Medical University, Harbin 150086, China

**Keywords:** Polymorphism, Bladder cancer, MTFHR, Ala222Val, Meta-analysis

## Abstract

**Background:**

The association between Methylenetetrahydrofolate reductase (MTHFR) Ala222Val (rs1801133) has been implicated to alter the risk of bladder cancer, but the results are controversial.

**Methods:**

A comprehensive databases of Pubmed, Embase, Web of Science, and the Chinese Biomedical Database (CBM) were searched for case–control studies investigating the association between MTHFR Ala222Val polymorphism and bladder cancer susceptibility. Odds ratios (OR) and 95% confidence intervals (95%CI) were used to assess this possible association. A *χ*^2^-based Q-test was used to examine the heterogeneity assumption. Begg’s and Egger’s test were used to examine the potential publication bias. The leave-one-out sensitivity analysis was conducted to determine whether our assumptions or decisions have a major effect on the results of the review. Statistical analysis was performed with the software program Stata 12.0.

**Results:**

A total of 15 independent studies were identified, including 3,570 cases and 3,926 controls. Our analysis suggested that Ala222Val was not associated with bladder cancer risk in overall population under additive model (OR=0.96, 95%CI=0.76-1.21, P=0.731), dominant model (OR=1.00, 95%CI=0.87-1.15, P=0.975), recessive model (OR=0.92, 95%CI=0.79-1.07, P=0.279), and Ala allele versus Val allele (OR=0.96, 95%CI=0.86-1.07, P=0.427). In the subgroup analysis stratified by ethnicity and sources of controls, there were also no significant associations detected among different descent populations, population-based studies and hospital-based studies.

**Conclusion:**

This meta-analysis showed the evidence that MTHFR Ala222Val polymorphism was not contributed to the development of bladder cancer.

**Virtual slide:**

The virtual slide(s) for this article can be found here: http://www.diagnosticpathology.diagnomx.eu/vs/2117182849994994.

## Introduction

Bladder cancer is the second most common genitourinary malignant disease, with an expected 73,510 newly diagnosed cases and 14,880 deaths in 2012 in America [1]. Many studies have suggested that several environmental factors such as cigarette smoking, aromatic amines contained in dyes, chronic inflammation due to infection, anticancer drugs and radiation are thought to be risk factors for bladder cancer [2]. However, most individuals with these known smoking habits, chemical or environmental exposures never develop bladder cancer while many bladder cancer cases develop among individuals without those known risk factors, suggesting that genetic factors also play an important role in bladder carcinogenesis [3]. Host factors including genetic polymorphisms, have been suggested as risk factors for the development of a variety of cancers, including bladder cancer.

Methylenetetrahydrofolate reductase (MTHFR) and methionine synthase (MS) are two enzymes essential for maintaining folate homeostasis. MTHFR catalyzes the irreversible conversion of 5,10-methylenetetrahydrofolate to 5-methyltetrahydrofolate, which serves as a substrate for the remethylation of homocysteine to methionine with subse-quent synthesis of S-adenosylmethionine (SAM). Polymorphisms in the methylenetetrahydrofolate reductase (MTHFR) gene are receiving increasing attention as variants that may potentially influence methyl group metabolism and, thereby, alter chromosome integrity. A common Ala222Val variant (an alanine to valine change) within the N-terminal catalytic domain results in a thermolabile enzyme with with 35-50% reduced activity [4]. Folate deficiency is associated with DNA strand breakage and uracil misincorporation into DNA [5]. Thus, if MTHFR polymorphic variants reduce folate levels by diminishing enzymatic activity, they could enhance the propensity for DNA strand breakage and cancer occurrence [6]. Alternatively, variant MTHFR activity could influence the availability of methyl donors by altering S-adeno-sylmethionine levels, and potentially, the methylation status of key tumor suppressor or promoter genes involved in bladder carcinogenesis [7]. Furthermore, functional polymorphisms in MTHFR could affect the metabolism of other carcinogenic substances that undergo one carbon metabolism such as arsenic [8,9].

Recently, many studies have investigated the role of the MTHFR Ala222Val polymorphism in the etiology of bladder cancer susceptibility. However, the results of these studies remain inconsistent. Therefore, we conducted a meta-analysis of all available case–control studies that have been published to assess the effect of the MTHFR Ala222Val polymorphism on the risk of bladder cancer.

## Methods

### Search strategy

We conducted a comprehensive search in the Pubmed, Embase, Web of Science, and Chinese Biomedical Database (CBM) databases updated on March 1, 2013. We combined search terms for MTHFR Ala222Val polymorphism and bladder cancer risk. Search terms included: (Methylene-tetrahydrofolate reductase, MTHFR, Ala222Val, or rs1801133) and bladder cancer. There was no sample size and language limitation. We evaluated all associated publications to retrieve the most eligible literatures. All references cited in the included studies were also hand-searched and reviewed to identify additional published articles not indexed in common databases. Of the studies with overlapping data published by the same authors, only the most recent or complete study was included in this meta-analysis.

### Inclusion and exclusion criteria

Studies included in this meta-analysis had to meet the following criteria: (1) evaluate the MTHFR Ala222Val polymorphism and bladder cancer risk, (2) Only the case–control studies were considered; (3) The paper should clearly describe the diagnoses of bladder cancer and the sources of cases and controls; (4) sufficient reported genotypic frequencies in both cases and controls for estimating an odds ratio (OR) with a 95% confidence interval (95%CI). The exclusion criteria were: (1) none case–control studies; (2) control population including malignant tumor patients; and (3) duplicated publications.

### Data extraction

Information was carefully extracted from all eligible publications independently by two investigators according to the selection criteria. In case of disagreement, a third reviewer assessed the articles. For each of the eligible case–control studies, the following data were collected: the first author’s last name, year of publication, ethnicity, source of the controls, total number of cases and controls, and genotype distributions in cases and controls.

### Quality assessment

Quality assessment of case–control studies in this meta-analysis was performed using the Newcastle Ottawa scale (NOS) as recommended by the Cochrane Non-Randomized Studies Methods Working Group [10]. This instrument was developed to assess the quality of nonrandomized studies, specifically cohort and case–control studies. Based on the NOS, case–control studies were judged based on three broad perspectives: selection of study groups (1 criterion), comparability of study groups (4 criteria), and ascertainment of outcome of interest (3 criteria). Given the variability in quality of observational studies found on our initial literature search, we considered studies that met 5 or more of the NOS criteria as high quality [10].

### Statistical analysis

We examined the association between MTHFR Ala222Val polymorphism and the bladder cancer risk by calculating pooled odds ratio (ORs) and 95% confidence intervals (95%CI). We explored the association between (Ala-allele vs Val-allele) comparison and bladder cancer development, as well as homozygote comparison (Ala/Ala versus Val/Val), dominant model [(Ala/Ala + Ala/Val) versus Val/Val)] and recessive model [(Ala/Ala versus Ala/Val + Val/Val)]. The significance of pooled OR was tested by Z test. The *χ*2-based Q-test was also used to examine the heterogeneity assumption [11]. If studies’ findings only differ by the sampling (P≥0.05), a fixed-effects model could be used to calculate the combined OR. By contrast, if the P value of the Q tests is below 0.05, which suggested that the study results statistically differ by heterogeneous case and sampling, a random-effects model could be more suitable. The significance of the pooled OR was determined by the Z-test, and P < 0.05 was considered as statistically significant.

The leave-one-out sensitivity analysis was conducted to determine whether our assumptions or decisions have a major effect on the results of the review by omitting each study [12]. Furthermore, subgroup analyses were performed to test whether the effect size varied by the ethnicity and the source of control population. To evaluate the published bias, we used Begg’s [13] and Egger’s [14] formal statistical test and by visual inspection of the funnel plot. All statistical analyses were performed with Stata software (version 12.0; Stata Corp LP, College Station, TX), using two-sided P values.

## Results

### Study characteristics

In total, 13 publications including 15 case–control studies with 3,570 cases and 3,926 controls met the selection criteria [15-27]. The characteristics of the studies included in this meta-analysis are listed in Table 1. Among the 15 studies, 5 studies of Caucasians, 5 studies of Asians, 4 studies of Africans and 1 study of mixed population, 8 studies of population-based controls, 7 studies of hospital-based controls. The distribution of the MTHFR Ala222Val genotype in the control groups of these 15 studies was all consistent with HWE (All *P*_*HWE*_ values were more than 0.05, Table 1). According to the quality criteria, all the 15 studies were high quality.

**Table 1:** Main characteristics of these studies included in this meta-analysis

**First author [Reference]**	**Year**	**Ethnicity**	**Sample size**		**Source of control**	**Cases**			**Controls**			**HWE**
						**Ala/Ala**	**Ala/Val**	**Val/Val**	**Ala/Ala**	**Ala/Val**	**Val/Val**	
			**case**	**control**								
Kimura [15]	2001	Caucasian	165	150	HB	70	80	15	65	73	12	0.17
Sanyal [16]	2004	Caucasian	309	246	PB	173	113	23	121	102	23	0.82
Lin ^1^[17]	2004	Caucasian	410	410	PB	176	183	51	196	164	50	0.09
Moore [18]	2004	Caucasian	106	109	PB	45	42	19	32	59	18	0.29
Lin ^2^[17]	2004	Mixed	17	17	PB	16	1	0	13	4	0	0.58
Lin ^3^[17]	2004	African	21	21	PB	7	13	1	9	9	3	0.76
Karagas [19]	2005	Caucasian	352	551	PB	140	171	39	227	245	71	0.70
Moore [20]	2006	Asian	1041	1049	PB	418	478	145	402	486	161	0.48
Ouerhani [21]	2007	African	111	131	HB	43	58	10	58	56	17	0.55
Wang [22]	2009	Asian	239	250	PB	66	129	45	88	132	30	0.07
Ouerhani [23]	2009	African	90	110	HB	33	50	7	51	45	14	0.42
Rouissi [24]	2009	African	185	191	HB	87	86	12	81	90	20	0.49
Cai [25]	2009	Asian	312	325	HB	82	169	61	113	170	42	0.08
Safarinejad [26]	2011	Asian	158	316	HB	67	74	17	144	142	30	0.56
Izmirli [27]	2011	Asian	54	50	HB	28	22	4	36	14	0	0.25

HB:Hospital-Based Study. PB: Population-Based Study. HWE: Hardy–Weinberg equilibrium. ^1,2,3^: Different descent populations in one publication.

### Quantitative synthesis

**Figure 1:**
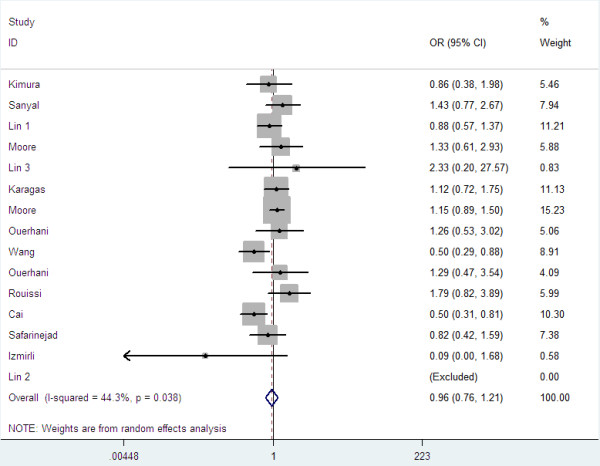
Forest plots for MTHFR Ala222Val polymorphism and risk of bladder cancer in overall populations (Ala/Ala versus Val/Val).

**Figure 2:**
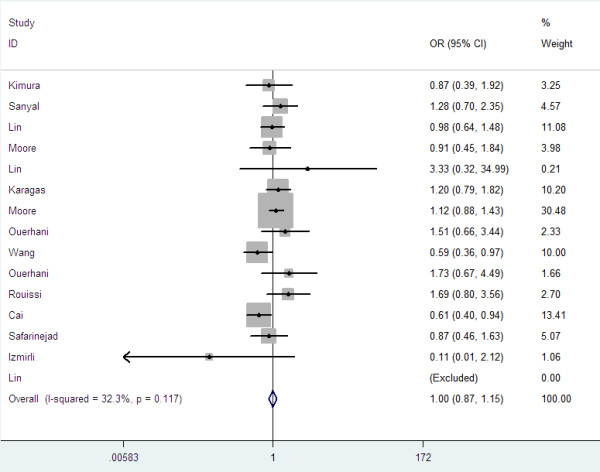
Forest plots for MTHFR Ala222Val polymorphism and risk of bladder cancer in overall populations (Dominant model).

**Figure 3:**
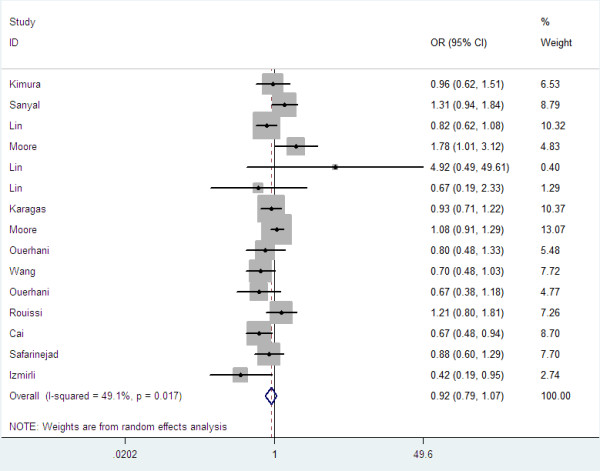
Forest plots for MTHFR Ala222Val polymorphism and risk of bladder cancer in overall populations (Recessive model).

**Figure 4:**
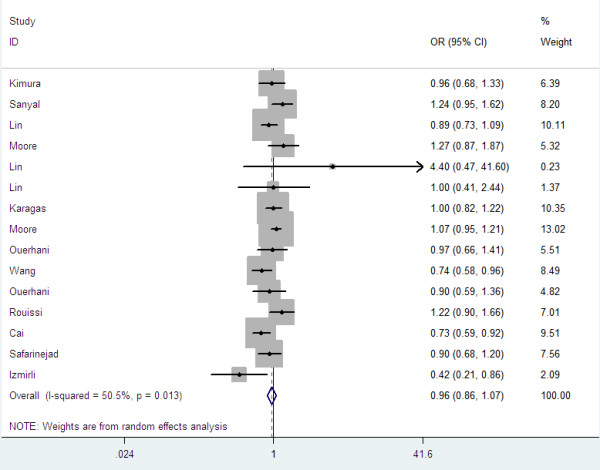
Forest plots for MTHFR Ala222Val polymorphism and risk of bladder cancer in overall populations (Ala allele vs Val allele).

Overall, there was no association between bladder cancer risk and the variant genotypes in different genetic models when all the eligible studies were pooled into the meta-analysis. As show in Table 2, compared with the wild-type homozygote genotype, the homozygote variant genotype Val/Val was not associated with a statistically significant increase risk of bladder cancer (OR=0.96, 95%CI=0.76-1.21, P=0.73, Figure 1). Simultaneously, no significant effects were observed in dominant (OR=1.00, 95%CI=0.87-1.47, P=0.96, Figure 2), recessive models (OR=0.92, 95%CI=0.79-1.07, P=0.28, Figure 3), and Ala allele vs Val allele comparison model (OR=0.96, 95%CI=0.86-1.07, P=0.43, Figure 4). When we analyzed the relationship of MTHFR Ala222Val polymorphism and bladder cancer risk in different ethnicity subgroup, our data showed that this polymorphism did not contribute to the bladder cancer risk among different descent populations. Subgroup analyses were also performed according to the source of control population. We didn’t observe any significant association both population-based study and hospital-based study in any genetic model using random effect model (Table 2).

**Table 2:** **Meta-analysis on the association between *MTHFR *Ala222Val polymorphism and bladder cancer risk**

**Variables**	**Study number**	**Sample size**		**Test of heterogeneity**		**Test of Association**	
		**case**	**control**	***P***_***h***_	***I***^***2***^	**OR(95% CL)**	***P***
**Ala/Ala vs Val/Val**							
Total	15	3570	3926	0.04	44.3%	0.96 (0.76 1.21)	0.73
**Ethnicity**							
Caucasian	5	1342	1466	0.70	0.0%	1.07 (0.83 1.38)	0.59
Asian	5	1804	1990	0.00	74.3%	0.68 (0.41 1.12)	0.13
African	4	407	453	0.91	0.0%	1.50 (0.91 2.45)	0.11
Mixed population	1	17	17	—	—	—	—
**Source of control**							
HB	7	1075	1273	0.07	49.3%	0.90 (0.58 1.39)	0.62
PB	8	2495	2653	0.14	37.5%	1.20 (0.79 1.31)	0.88
**Ala/Ala vs Ala/Val**							
Total	15	3570	3926	0.05	41.3%	0.92 (0.80 1.06)	0.24
**Ethnicity**							
Caucasian	5	1342	1466	0.05	58.9%	1.05 (0.81 1.37)	0.70
Asian	5	1804	1990	0.15	40.4%	0.86 (0.69 1.06)	0.15
African	4	407	453	0.26	25.9%	0.80 (0.56 1.13)	0.20
Mixed population	1	17	17	—	—	4.92 (0.49 49.61)	0.18
**Source of control**							
HB	7	1075	1273	0.40	3.7%	0.82 (0.68 0.98)	0.03
PB	8	2495	2653	0.04	51.5%	1.01 (0.82 1.23)	0.94
**Ala/Val vs Val/Val**							
Total	15	3570	3926	0.22	21.5%	1.02 (0.85 1.23)	0.81
**Ethnicity**							
Caucasian	5	1342	1466	0.69	0.0%	1.07 (0.83 1.37)	0.60
Asian	5	1804	1990	0.18	36.0%	0.84 (0.63 1.12)	0.24
African	4	407	453	0.86	0.0%	1.86 (1.14 3.03)	0.01
Mixed population	1	17	17	—	—	—	—
**Source of control**							
HB	7	1075	1273	0.14	38.6%	1.08 (0.73 1.59)	0.71
PB	8	2495	2653	0.35	10.7%	1.03 (0.85 1.25)	0.76
**Dominant model**							
Total	15	3570	3926	0.12	32.3%	1.00 (0.87 1.15)	0.98
**Ethnicity**							
Caucasian	5	1342	1466	0.86	0.0%	1.07 (0.84 1.35)	0.58
Asian	5	1804	1990	0.03	63.4%	0.77 (0.52 1.12)	0.17
African	4	407	453	0.94	0.0%	1.68 (1.05 2.69)	0.03
Mixed population	1	17	17	—	—	—	—
**Source of control**							
HB	7	1075	1273	0.09	46.0%	1.00 (0.67 1.49)	0.99
PB	8	2495	2653	0.29	18.4%	1.03 (0.84 1.25)	0.80
**Recessive model**							
Total	15	3570	3926	0.02	49.1%	0.92 (0.79 1.07)	0.28
**Ethnicity**							
Caucasian	5	1342	1466	0.07	54.4%	1.06 (0.83 1.34)	0.66
Asian	5	1804	1990	0.02	67.0%	0.79 (0.60 1.04)	0.09
African	4	407	453	0.33	12.9%	0.90 (0.67 1.21)	0.49
Mixed population	1	17	17	—	—	4.92 (0.49 49.61)	0.18
**Source of control**							
HB	7	1075	1273	0.20	29.8%	0.92 (0.66 1.01)	0.06
PB	8	2495	2653	0.04	53.5%	1.01 (0.83 1.23)	0.90
**Ala-allele vs Val-allele**							
Total	15	3570	3926	0.01	50.5%	0.96 (0.86 1.07)	0.43
**Ethnicity**							
Caucasian	5	1342	1466	0.26	23.8%	1.03 (0.90 1.18)	0.66
Asian	5	1804	1990	0.00	76.5%	0.81 (0.65 1.03)	0.08
African	4	407	453	0.65	0.0%	1.05 (0.86 1.29)	0.63
Mixed population	1	17	17	—	—	4.40 (0.47 41.60)	0.20
**Source of control**							
HB	7	1075	1273	0.07	48.0%	0.89 (0.74 1.06)	0.19
PB	8	2495	2653	0.07	47.3%	1.01 (0.89 1.15)	0.89

*HB *Hospital-Based Study, *PB *Population-Based Study, *P*_*h *_P value of heterogeneit.

### Sensitivity analysis

**Figure 5:**
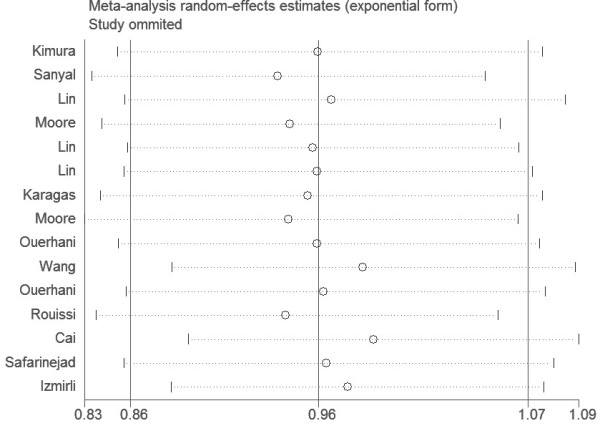
Sensitive analysis for the MFHTR Ala222Val polymorphism illustrating the influence of each studies on pooled OR (Ala- allele vs Val allele).

In order to compare the sensitivity of this meta-analysis, we conducted a leave-one-out sensitivity analysis. A single study involved in this meta-analysis was evaluated each time to reflect the influence of the individual data set to pooled ORs. The results pattern was not impacted by single study in all genetic models, for example, under the allele contrast model (Ala versus Val) was shown in Figure 5. The P for Q test and the I^2^ value also showed that none of single study affected the heterogeneity of this meta-analysis.

### Publication bias

**Figure 6:**
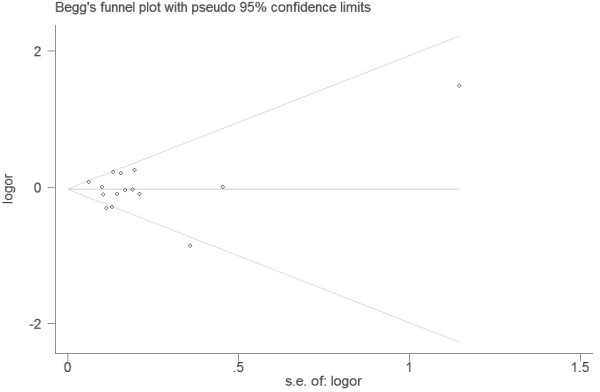
Begg’s funnel plot for assessing the publication bias under the allele contrast model (Ala allele vs. Val allele).

**Figure 7:**
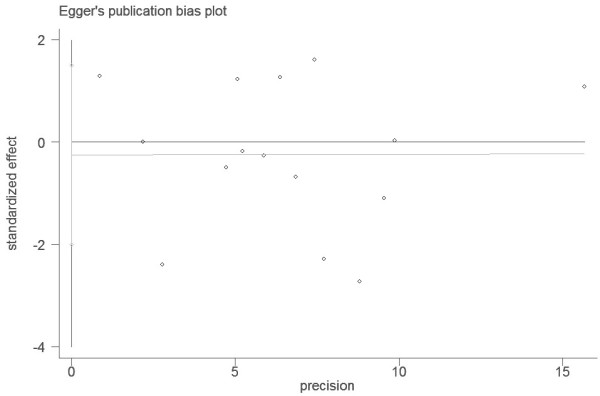
Egger’s funnel plot for assessing the publication bias under the allele contrast model (Ala allele vs. Val allele).

Begg’s funnel plot and Egger’s test were used to assess the publication bias in this meta-analysis. The Funnel plots’ shape of all contrasts did not reveal obvious evidence of asymmetry, and all the P values of Egger’s test were more than 0.05, providing statistical evidence for the funnel plots’ symmetry. For example, this meta-analysis investigated the association between MTHFR Ala222Val polymorphism and bladder cancer under the allele contrast model (Ala versus Val). Begg’s test for the allele contrast model was shown in Figure 6, and Egger’s test for the allele contrast model was shown in Figure 7. Thus, the above results suggest that publication bias was not evident in this meta-analysis.

## Discussion

It is wide acknowledged that genetics play an important role in determining cancer risk and association studies have been identified to evaluate cancer susceptibility [28]. However, many associated studies failed to provide convincing evidence of linkage and resulted in contradicting findings. Meta-analysis provided a popular method for combining world literatures across studies to resolve the statistical power and discrepancy problem in associate studies [29]. It was more systematic for its statistical methods than single case–control studies and cohort studies [30-32].

Previous epidemiological studies have evaluated the association between bladder cancer risk and MTHFR Ala222Val polymorphism, but with inconclusive results. An initial cohort study suggested a 5.5 fold risk of bladder and kidney cancer combined among 222 Ala>Val variant homozygotes [33]. In contrast, three case–control studies provided the evidence that there was no overall association of MTHFR Ala222Val genotype with bladder cancer [15-17], one study observed a reduced risk among heterozygotes for MTHFR Ala222val [18].

Based on 15 studies providing data on MTHFR Ala222Val polymorphism and bladder cancer risk, we conduced a meta-analysis involving in 3,570 cases and 3,926 controls to indicate if this polymorphism was significantly associated with bladder cancer risk. Moreover, we also evaluated the publication bias. The MTHFR Ala222Val genotypes funnel plot was approximately symmetrical. Furthermore, Begg’s and Eggle’s test showed that there was no publication bias in this study. We found that there were no significant associations between this polymorphism and bladder cancer risk in the all genetic models. These results suggested that the MTHFR Ala222Val polymorphism might be not contributed to the development of bladder cancer. It has been well known that cancer occurrence and mortality varied by ethnicity and geographic location [34]. In this meta-analysis, all subjects were sub-grouped into four groups (Caucasian, Asian, African and mixed populations). No association of Ala222Val polymorphism with bladder cancer risk was detected in different descent populations. When stratified by source of controls, the similar results were found both in population-based and hospital-based studies.

Some limitations of our meta-analysis should be addressed. Firstly, the numbers of published studies collected in our analysis were not large enough for the comprehensive analysis. Secondly, lacking the original data of the included studies limited our study to further evaluate the potential interactions, since gene-environment and gene-gene interactions may modulate bladder cancer risk. So, a more precise analysis needs to be conducted if individual data such as age and sex are available. Nevertheless, advantages in our meta-analysis should also be acknowledged. A systematic review of the association of MTHFR Ala222Val polymorphism with bladder cancer risk is statistically more powerful than any single study. Furthermore, the studies included in our meta-analysis strictly and satisfactory met our selection criteria.

## Conclusions

In conclusion, our meta-analysis provided the evidence that the MTHFR Ala222Val polymorphism maybe not contributed to the development of bladder cancer. In addition, further studies with larger sample sizes and careful design are needed to identify this association more comprehensively.

## Competing interests

None of the authors have any competing interests to declare.

## Authors’ contributions

WX, HZ, and FW carried out the meta-analysis study, drafted the manuscript and involved in revising the manuscript critically for important intellectual content. HZ and FW participated in the design of the study and revised the manuscript. WX carried out the meta-analysis study and drafted the manuscript. HW participated in the design of the study, drafted the manuscript and revised the manuscript. All authors read and approve the final manuscript.

## References

[B1] Siegel R Naishadham D Jemal A Cancer statistics, 2012 CA Cancer J Clin 2012 62 10 29 10.3322/caac.20138 22237781

[B2] Hirao Y Kim WJ Fujimoto K Environmental factors promoting bladder cancer Curr Opin Urol 2009 19 494 499 10.1097/MOU.0b013e32832eb4ef 19553820

[B3] Taioli E Raimondi S Genetic susceptibility to bladder cancer Lancet 2005 366 610 612 10.1016/S0140-6736(05)67115-2 16112283

[B4] Frosst P Blom HJ Milos R Goyette P Sheppard CA Matthews RG Boers GJ Den Heijer M Kluijtmans LA Van Den Heuvel LP Rozen R A candidate genetic risk factor for vascular disease: a common mutation in methylenetetrahydrofolate reductase Nat Genet 1995 10 111 113 10.1038/ng0595-111 7647779

[B5] Duthie SJ Narayanan S Brand GM Pirie L Grant G Impact of folate deficiency on DNA stability J Nutr 2002 132 2444S 2449S 12163709 10.1093/jn/132.8.2444S

[B6] Bailey LB Folate, methyl-related nutrients, alcohol, and the MTHFR 677C4T polymorphism affect cancer risk: intake recommendations J Nutr 2003 133 3748S 3753S 14608109 10.1093/jn/133.11.3748S

[B7] Duthie SJ Narayanan S Blum S Pirie L Brand GM Folate deficiency in vitro induces uracil misincor-poration and DNA hypomethylation and inhibits DNA excision repair in immortalized normal human colon epithelial cells Nutr Cancer 2000 37 245 251 10.1207/S15327914NC372_18 11142099

[B8] McDorman EW Collins BW Allen JW Dietary folate deficiency enhances induction of micronuclei by arsenic in mice Environ Mol Mutagen 2002 40 71 77 10.1002/em.10085 12211079

[B9] Spiegelstein O Lu X Le XC Troen A Selhub J Melnyk S James SJ Finnell RH Effects of dietary folate intake and folate binding protein-1 (Folbp1) on urinary speciation of sodium arsenate in mice Toxicol Lett 2003 145 167 174 10.1016/S0378-4274(03)00307-2 14581169

[B10] Wells GA Shea B O'Connell D The Newcastle–Ottawa Scale (NOS) for assessing the quality of non-randomised studies in meta-analyses 2011 Available at: http://www.ohri.ca/programs/clinical_epidemiology/oxford.asp. Accessed February 22, 201123888427

[B11] DerSimonian R Laird N Meta-analysis in clinical trials Control Clin Trials 1986 7 3 177 188 10.1016/0197-2456(86)90046-2 3802833

[B12] Hysong SJ Meta-analysis: audit and feedback features impact effectiveness on care quality Med Care 2009 47 3 356 363 10.1097/MLR.0b013e3181893f6b 19194332 PMC4170834

[B13] Begg CB Mazumdar M Operating characteristics of a rank correlation test for publication bias Biometrics 1994 50 4 1088 1101 10.2307/2533446 7786990

[B14] Egger M Davey Smith G Schneider M Minder C Bias in meta-analysis detected by a simple, graphical test Bmj 1997 315 7109 629 634 10.1136/bmj.315.7109.629 9310563 PMC2127453

[B15] Kimura F Florl AR Steinhoff C Golka K Willers R Seifert HH Schulz WA Polymorphic methyl group metabolism genes in patients with transitional cell carcinoma of the urinary bladder Mutat Res 2001 458 49 54 10.1016/S1383-5726(01)00010-3 11406421

[B16] Sanyal S Festa F Sakano S Zhang Z Steineck G Norming U Wijkstrom H Larsson P Kumar R Hemminki K Polymorphisms in DNA repair and metabolic genes in bladder cancer Carcinogenesis 2004 25 729 734 14688016 10.1093/carcin/bgh058

[B17] Lin J Spitz MR Wang Y Schabath MB Gorlov IP Hernandez LM Pillow PC Grossman HB Wu X Polymorphisms of folate metabolic genes and susceptibility to bladder cancer: a case–control study Carcinogenesis 2004 25 1639 1647 10.1093/carcin/bgh175 15117811

[B18] Moore LE Wiencke JK Bates MN Zheng S Rey OA Smith AH Investigation of genetic poly-morphisms and smoking in a bladder cancer case–control study in Argentina Cancer Lett 2004 211 199 207 10.1016/j.canlet.2004.04.011 15219943

[B19] Karagas MR Park S Nelson HH Methylenetetrahydrofolate reductase (MTHFR) variants and bladder cancer: a population-based case–control study Int J Hyg Environ Health 2005 208 321 327 10.1016/j.ijheh.2005.04.005 16217917

[B20] Moore LE Malats N Rothman N Polymorphisms in one-carbon metabolism and trans-sulfuration pathway genes and susceptibility to bladder cancer Int J Cancer 2007 120 2452 2458 10.1002/ijc.22565 17311259

[B21] Ouerhani S Oliveira E Marrakchi R Methylenetetrahydrofolate reductase and methionine synthase polymorphisms and risk of bladder cancer in a Tunisian population Cancer Genet Cytogenet 2007 176 48 53 10.1016/j.cancergencyto.2007.03.007 17574963

[B22] Wang M Zhu H Fu G Polymorphisms of methylenetetrahydrofolate reductase and methionine synthase genes and bladder cancer risk: a case–control study with meta-analysis Clin Exp Med 2009 9 9 19 10.1007/s10238-008-0013-1 18815869

[B23] Ouerhani S Rouissi K Marrakchi R Combined effect of NAT2, MTR and MTHFR genotypes and tobacco on bladder cancer susceptibility in Tunisian population Cancer Detect Prev 2009 32 395 402 10.1016/j.canep.2009.04.005 19588544

[B24] Rouissi K Ouerhani S Oliveira E Polymorphisms in one-carbon metabolism pathway genes and risk for bladder cancer in a Tunisian population Cancer Genet Cytogenet 2009 195 43 53 10.1016/j.cancergencyto.2009.06.007 19837268

[B25] Cai DW Liu XF Bu RG Chen XN Genetic polymorphisms of MTHFR and aberrant promoter hypermethylation of the RASSF1A gene in bladder cancer risk in a Chinese population J Int Med Res 2009 37 1882 1889 10.1177/147323000903700625 20146887

[B26] Safarinejad MR Shafiei N Safarinejad S Genetic susceptibility of methylenetetrahydrofolate reductase (MTHFR) gene C677T, A1298C, and G1793A polymorphisms with risk for bladder transitional cell carcinoma in men Med Oncol 2011 28 1 S398 S412 21046286 10.1007/s12032-010-9723-9

[B27] Izmirli M Inandiklioglu N Abat D Alptekin D Demirhan O Tansug Z Bayazit Y MTHFR gene polymorphisms in bladder cancer in the Turkish population Asian Pac J Cancer Prev 2011 12 1833 1835 22126575

[B28] Risch N Merikangas K The future of genetic studies of complex human diseases Science 1996 273 5281 1516 1517 10.1126/science.273.5281.1516 8801636

[B29] Munafo MR Flint J Meta-analysis of genetic association studies Trends Genet 2004 20 9 439 444 10.1016/j.tig.2004.06.014 15313553

[B30] Liwa Y Jianqiu C Association of MHTFR Ala222Val (rs1801133) polymorphism and breast cancer susceptibility: An update meta-analysis based on 51 research studies Diagnostic Pathology 2012 7 171 10.1186/1746-1596-7-171 23217001 PMC3536596

[B31] Xue Q Qiliu P Aiping Q Zhiping C Liwen L Yan D Li X Juanjuan X Haiwei L Taijie L Shan L Jinmin Z Association of COMT Val158Met polymorphism and breast cancer risk: an updated meta-analysis Diagnostic Pathology 2012 7 136 10.1186/1746-1596-7-136 23039364 PMC3543196

[B32] de Matos L Adriana Del G Carolina M de Lima Farah M Auro Del G da Silva Pinhal M Expression of ck-19, galectin-3 and hbme-1 in the differentiation of thyroid lesions: systematic review and diagnostic meta-analysis Diagn Pathol 2012 7 97 10.1186/1746-1596-7-97 22888980 PMC3523001

[B33] Heijmans BT Boer JM Suchiman HE Cornelisse CJ Westendorp RG Kromhout D Feskens EJ Slagboom PE A common variant of the methyle-netetrahydrofolate reductase gene (1p36) is associated with an increased risk of cancer Cancer Res 2003 63 1249 1253 12649184

[B34] Gilliland FD Ethnic differences in cancer incidence: a marker for inherited susceptibility? Environ Health Perspect 1997 105 Suppl 4 897 900 10.1289/ehp.97105s4897 9255577 PMC1470049

